# Supplementary material for: How does a pair of near-vision spectacle correction empower older Zanzibari craftswomen?: A qualitative study on perception
Source: PLoS One. 2023 May 26;18(5):e0286315. doi: 10.1371/journal.pone.0286315 (PMC10218727; doi:10.1371/journal.pone.0286315)
Supplement: S1 File — (DOCX) [file pone.0286315.s001.docx]

S1 File: Inclusivity in global research checklist

**Ethical considerations, permits and authorship**

Provide details as to who granted permissions and/or consent for the study to take place in the Methods section of your manuscript. This should include the names of **all** ethics boards, governmental organizations, community leaders or other bodies that provided approval for the study. If individuals provided approval refer to these people by their role or title but do not list their name(s).

Reported on page number: 9

This study received approvals from the Ethics Committees from the Zanzibar Human Research Institute (ZAHREC/04/PR/MARCH/2022/12), Zanzibar Office of Government Chief Statistician (6221C2601263D) and Queen's University Belfast (MHLS 22_72). We obtained the participants' informed consent before the interviews were conducted.

If there were any deviations from the study protocol after approval was obtained please provide details of these changes in the Methods section of your manuscript.
Did this study involve local collaborators that are residents of the country where the research was conducted or members of the community studied? If you do not have any authors from said communities, please provide an explanation for this below.

Yes. FO, OO and EM are local collaborators that are residents of Tanzania (where the research was conducted).

Reported on page number: Not applicable

Everyone listed as an author should meet PLOS’ criteria for authorship and all individuals who meet these criteria should be included in the author byline, rather than the acknowledgements. Authorship criteria is based on the International Committee of Medical Journal Editors (ICMJE) Uniform Requirements for Manuscripts Submitted to Biomedical Journals - for further information please see here: <https://journals.plos.org/plosone/s/authorship>.

**Human subjects research (e.g. health research, medical research, cross-cultural psychology)**

Did you obtain written informed consent from a representative of the local community or region before the research took place? How did you establish who speaks for the community? Details of written informed consent obtained from study participants should be reported separately in the Methods section of your manuscript.

Yes. We conducted a consultation meeting prior to the development of the project. We identified that the gatekeepers to be contacted were the women cooperative leaders that hosted the craftswomen.

How did members of the local community provide input on the aims of the research investigation, its methodology, and its anticipated outcome(s)?

FO and OO invited the leaders and craftswomen to the Theory of Change workshop, which included a session of briefing, development of the study protocol and data collection schedule.

When engaging with the local community, how did you ensure that the informed consent documents and other materials could be understood by local stakeholders?

All informed consent documents were drafted by FO, OO, EM and reviewed by CG ( a Swahili-speaking anthropologist) and a sample of 20 craftswomen. Amendments were made based on their feedback.

Will the findings of the research be made available in an understandable format to stakeholders in the community where the study was conducted (e.g. via a presentation, summary report, copies of publications, etc.)? Please provide details of how this will be achieved.

Yes. A dissemination event will be conducted in April 2023 to the local stakeholders and craftswomen. A lay summary report will be developed and shared with the local stakeholders. All publications will be made available in an open-access manner, emailed to the leaders and include on the WE-ZACE website.

**Non-human subjects research using specimens/ animals collected as part of the study, or those housed in archival collections. Examples include archaeology, paleontology, botany and zoology.**

Did the permission you obtained from a local authority to perform the study include an agreement on access to outputs and benefit sharing? This may include procedures to enable fair distribution of the benefits and resources arising from the research performed. Please include any details of Prior Informed Consent and Benefit Sharing Agreements obtained. These may be required by field-specific regulations, for example the Convention on Biological Diversity (CBD) and the associated Nagoya Protocol.

Not applicable

If the material used in your study was imported, please A) provide the year it was imported and B) indicate whether permits were obtained to import/export the materials used, C) provide details of any permits obtained. If this information is not available, please indicate this.

Not applicable

If you used archival specimens, please state how the material used in your study was acquired by the institute it is held in and provide details of any permits obtained for the original excavations/ sample collection. If this information is not available, please indicate this.

Not applicable

How was the potential cultural significance of the materials collected in your study to local communities considered in your research design? Were Indigenous peoples and/or local researchers and institutions involved with archaeological excavations / collection of specimens? If so, please provide a description of their involvement.

Not applicable

If your manuscript includes photographs of human remains please indicate whether authors obtained permission from descendants or affiliated cultural communities to do so.

Not applicable
