# Supplementary material for: How does a pair of near-vision spectacle correction empower older Zanzibari craftswomen?: A qualitative study on perception
Source: PLoS One. 2023 May 26;18(5):e0286315. doi: 10.1371/journal.pone.0286315 (PMC10218727; doi:10.1371/journal.pone.0286315)
Supplement: Attachment — Submitted filename: Response to reviewers.docx [file pone.0286315.s002.docx]

Journal requirements:

When submitting your revision, we need you to address these additional requirements.

**1. Please ensure that your manuscript meets PLOS ONE’s style requirements, including those for file naming. The PLOS ONE style templates can be found at**

[**https://journals.plos.org/plosone/s/file?id=wjVg/PLOSOne_formatting_sample_main_body.pdf**](https://journals.plos.org/plosone/s/file?id=wjVg/PLOSOne_formatting_sample_main_body.pdf) **and**

[**https://journals.plos.org/plosone/s/file?id=ba62/PLOSOne_formatting_sample_title_authors_affiliations.pdf**](https://journals.plos.org/plosone/s/file?id=ba62/PLOSOne_formatting_sample_title_authors_affiliations.pdf)

Response: Noted with changes made.

**2. Please include a complete copy of PLOS’ questionnaire on inclusivity in global research in your revised manuscript. Our policy for research in this area aims to improve transparency in the reporting of research performed outside of researchers’ own country or community. The policy applies to researchers who have travelled to a different country to conduct research, research with Indigenous populations or their lands, and research on cultural artefacts. The questionnaire can also be requested at the journal’s discretion for any other submissions, even if these conditions are not met.  Please find more information on the policy and a link to download a blank copy of the questionnaire here:** [**https://journals.plos.org/plosone/s/best-practices-in-research-reporting**](https://journals.plos.org/plosone/s/best-practices-in-research-reporting)**. Please upload a completed version of your questionnaire as Supporting Information when you resubmit your manuscript.**

Response: We have now uploaded the completed form as Supporting Information.

3. Please provide additional details regarding participant consent. In the ethics statement in the Methods and online submission information, please ensure that you have specified what type you obtained (for instance, written or verbal, and if verbal, how it was documented and witnessed). If your study included minors, state whether you obtained consent from parents or guardians. If the need for consent was waived by the ethics committee, please include this information.

**4. Thank you for stating the following in the Competing Interests section:**

**“MFM.**

**This study formed part of MFM’s master’s dissertation.”**

**Please confirm that this does not alter your adherence to all PLOS ONE policies on sharing data and materials, by including the following statement: “This does not alter our adherence to  PLOS ONE policies on sharing data and materials.” (as detailed online in our guide for authors** [**http://journals.plos.org/plosone/s/competing-interests**](http://journals.plos.org/plosone/s/competing-interests)**).  If there are restrictions on sharing of data and/or materials, please state these. Please note that we cannot proceed with consideration of your article until this information has been declared.**

**Please include your updated Competing Interests statement in your cover letter; we will change the online submission form on your behalf.**

Response: We have included the additional clause in the manuscript and cover letter.

**5. Thank you for stating the following in the Acknowledgments Section of your manuscript:**

**“We want to thank the Novartis (Excellence in Ophthalmology and Vision Award, XOVA) funded the project [grant number NPO 6240 R8898CPH]. The funder has not contributed to the design and the analysis of the study.”**

**We note that you have provided funding information that is currently declared in your Funding Statement. However, funding information should not appear in the Acknowledgments section or other areas of your manuscript. We will only publish funding information present in the Funding Statement section of the online submission form.**

**Please remove any funding-related text from the manuscript and let us know how you would like to update your Funding Statement. Currently, your Funding Statement reads as follows:**

**“YES.**

**VFC.**

**NPO 6240 R8898CPH**

**Novartis (Excellence in Ophthalmology and Vision Award, XOVA)**

[**https://www.xova.novartis.com/#dwell**](https://www.xova.novartis.com/#dwell)

**The funder did not play any role in the study design, data collection and analysis, decision to publish, or preparation of the manuscript”**

**Please include your amended statements within your cover letter; we will change the online submission form on your behalf.**

Response: Amended.

**6. We note that you have stated that you will provide repository information for your data at acceptance. Should your manuscript be accepted for publication, we will hold it until you provide the relevant accession numbers or DOIs necessary to access your data. If you wish to make changes to your Data Availability statement, please describe these changes in your cover letter and we will update your Data Availability statement to reflect the information you provide.**

Response: We have now provided the doi number and the link to the dataset. (https://doi.org/10.17034/d32d197f-87fa-4c4c-bc08-b80e2e1c366d)

**7. Please review your reference list to ensure that it is complete and correct. If you have cited papers that have been retracted, please include the rationale for doing so in the manuscript text, or remove these references and replace them with relevant current references. Any changes to the reference list should be mentioned in the rebuttal letter that accompanies your revised manuscript. If you need to cite a retracted article, indicate the article’s retracted status in the References list and also include a citation and full reference for the retraction notice.**

Response: We have checked the reference list to ensure its completeness and accuracy.

Additional Editor Comments:

Dear Authors
Thank you for your submission. This a good manuscript in its field. I have outlined the comments provided by reviewers.

Reviewer 1:
I have mentioned few suggestions below:
Abstract
**1. Keywords: I suggest authors to arrange in alphabetical order.**

Response: Amended.

**2. Introduction: In line 60 and 78, kindly check the intext citation format. It should be similar. Also, in text citation should be in serial order.**

Response: Amended. We have standardise the citation format and made sure they are in serial order.

Reviewer 2
**Line 28: Kindly include your study duration.**
Response: Yes. Included study duration in Line 28.

**Line 40: You can add your major finding as per your objective.**

Response: Amended to include the major finding (Older craftswomen perceived that correcting near vision could empower them at personal and relational levels that encompass economic, psychological, social, political and educational empowerment).

**Line 46-123: Gross comment for Introduction section: Be more specific as per your objective. You can rearrange many points included in the introduction section to methodology section. It seems better. Writing all the methodological details will misguide the readers and they might be not interested. So, be specific in your introduction. Better to use 125 words.**

Response: Thank you for the comment. We streamlined the introduction section by removing information that might be too much for the readers and moving some content to the methods section. We also ensured that the flow of the content is logical and relevant to the subject matter.

**Line 125: add study duration, you need to start with type of research design, approach etc not from ethical approval/ You need restructuring of your method section.**

Response: We have included the study duration in Line 165. We have restructured the methods section as best as possible by balancing Reviewer 2’s comments, PLoS ONE’s guidelines, the COREQ checklist and sample PLoS ONE publications on similar qualitative research.

**Line 195: Result section has few typo errors, kindly correct. Somewhere the syntax is not maintained. Kindly improve your syntax.**

Response: Thank you for pointing out the typos and syntax errors. They are now amended.

**In your discussion, you need to add other social, political and economic factors that might be confounding variables. You need cautious interpretation.**

Response: Thank you very much for this excellent point. Indeed, the visual map (previously as supplementary material, now made into Figure 1 as part of the manuscript) shows that the pathway to women’s empowerment via corrected near vision could be both straightforward and complex. The complexity is seen as the interactions between all the factors mentioned by Reviewer 2 that could have confounded and/or led to the perceptions of different aspects of empowerment. We included a paragraph explaining this.

**In your conclusion: better not to write a conclusion which was beyond your scope of research. Actually, that needs further research.**
Response: Thank you for the comment. We now streamlined the conclusion to only include the main findings, the implication of the findings and the need for further research.

**In your reference, some typo errors noted, kindly rectify.**

Response: Amended.
